# Supplementary material for: Uncovering feature interdependencies in high-noise environments with stepwise lookahead decision forests
Source: Sci Rep. 2021 Apr 29;11:9238. doi: 10.1038/s41598-021-88571-3 (PMC8085031; doi:10.1038/s41598-021-88571-3)
Supplement: Supplementary file 1 — Supplementary Information. [file 41598_2021_88571_MOESM1_ESM.pdf]

Supplementary Information:
Uncovering Feature Interdependencies in High-Noise Environments with Stepwise Lookahead Decision Forests

SI Section 1: More Tests with Synthetic Data

In the main paper, we have coupled the first two features to the target variable in an XOR-junction, and accompanied them with six random noise features. To make sure that our results are not crucially dependent on this specific set-up, we have run some additional tests. In practical applications, it is unrealistic to assume that there is only two XOR-like features and no additional signal. We thus make the synthetic data more realistic and consider the same $N = 2,000$ samples from above, but now include six additional features $F^{(2)}, \ldots, F^{(7)}$ that are 0.1ρ Pearson correlated with the target. These features act to obfuscate the still highly structured XOR-type pattern. Figure SI 1 depicts the outcome of this result. Accordingly, compared to the previous example with XOR and noise features, the classification performances of the GRF, GDT, and XGB slightly increase, while the performance of the LRF remains essentially unchanged. Thus, while the gap between classification performance decreases when traditional linear signal is added to the data, the LRF still retains superior accuracy.

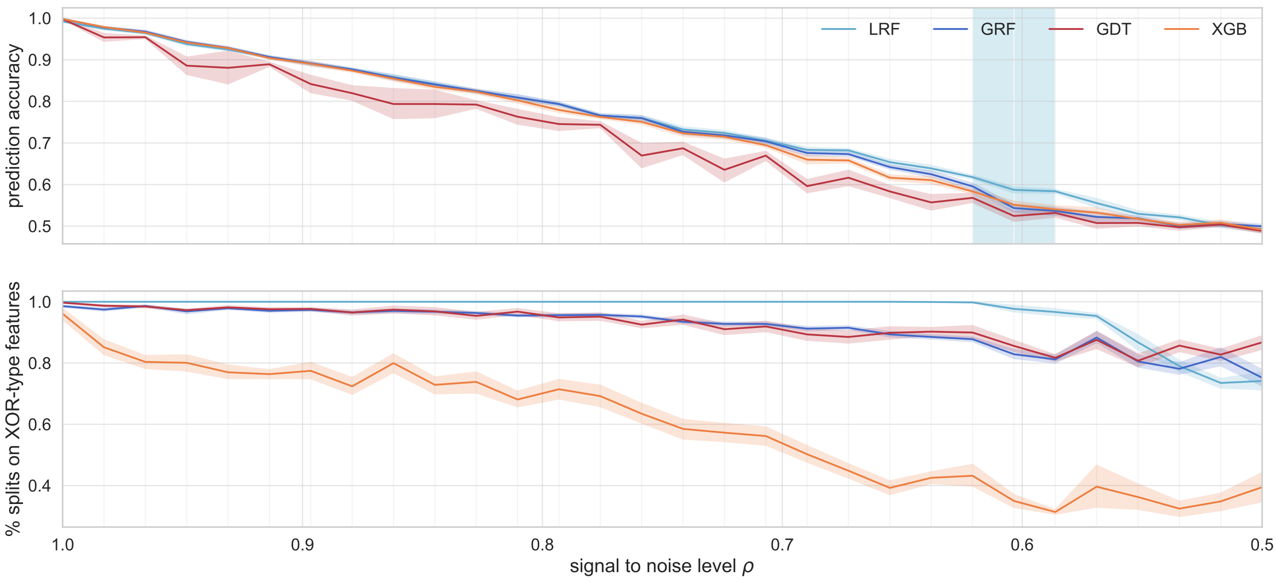

Figure SI 1: Same setup as Figure 2 in the main paper, but instead of six random noise feature, we add six features that are weakly linearly correlated with the target. See text for an interpretation.

Finally, we consider a synthetic dataset samples with the XOR-type relationship encoded $F^{(0)}$ and $F^{(1)}$ removed. The $N = 2,000$ samples are now composed of only 8 features $F^{(2)}, \ldots, F^{(7)}$, all of which are correlated with the target at $0.5 * (\rho - 0.5)$. We once again train LRF, GRF, GDT, and XGB models. Upon running multiple iterations of the above simulation, we find that both the resulting

GRF and LRF models consistently demonstrate almost equivalent prediction accuracies. A binomial test with a p-value well below 1% confirms that neither the LRF nor the other models consistently outperforms the others.

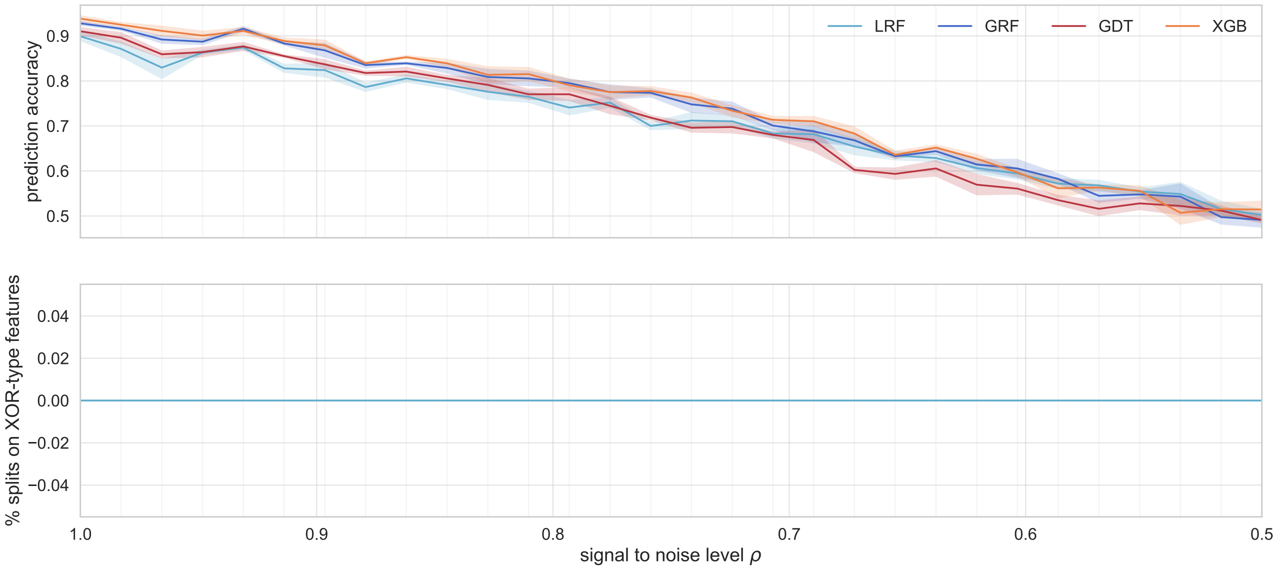

Figure SI 2: Same setup as Figure 2 in the main paper, but only with six linearly correlated features and no XOR-like dependencies. See text for an interpretation.

SI Section 2: Definition of Hyperparameters

The number of DTs that make up the RF can be considered a hyperparameter, referred to as T in the main paper. The parameter T should be chosen large enough to exhaust all different feature combinations. Picking an even larger number of trees is not beneficial, but also not detrimental to performance [1]. Through this article, we have thus set the number of decision trees equal to $2,000$ for both the LRF and the GRF. This is be sufficient to exhaust all combinations among the maximum 8 features that we consider. The remaining hyperparameters are determined by means of a 5-fold cross-validation.

LRF Hyperparameters

For the stepwise lookahead random forest (LRF), we have tuned the following parameters:

max depth This is the maximum depth that any DT can have. We test for $2, 4, 10$ or no restriction at all. We found that a limited depth of 2 was consistently the selected parameter, indicating the tendency of deeper trees to overfit, in particular in the noise data that was subject to our study.

min samples leaf This is the minimum fraction of data points that must be allocated to any of the terminal leaves. We test for $2\%, 10\%$ or no restriction at all.

max feat This is the number of features $\tilde{k}$ that are randomly selected from the total $k = 8$ features for any given node to split on. We test for $\tilde{k} = k$ and $\tilde{k} = k/2$.

As mentioned in the main paper, another hyperparameter is the number of considered split values B. While this could be treated as a hyperparameter, or also as a trade-off between precision and

execution time, here we have set it equal to the number of datapoints N throughout all applications. We have checked that setting this value equal to $N/2$ did not noticeably decrease the accuracy of our results. The value could likely be decreased even further, if computational time ought to be an issue. We also reference various efficient approximation methods in the main paper.

GRF Hyperparameters

For the vanilla, greedy random forest (GRF), we have tuned the following parameters:

max depth This is the maximum depth that any DT can have. We test for $2, 4, 10$ or no restriction at all.

min samples leaf This is the minimum fraction of data points that must be allocated to any of the terminal leaves. We test for $2\%, 10\%$ or no restriction at all.

max feat This is the number of features $\tilde{k}$ that are randomly selected from the total $k = 8$ features for any given node to split on. We test for $\tilde{k} = k = 8$ and $\tilde{k} = k/2 = 4$.

LDT Hyperparameters

For the single, stepwise lookahead decision tree (LDT) we use the same hyperparameters as for the LRF, with the exception of "max feat".

GDT Hyperparameters

For the vanilla, greedy decision tree (GDT), we have tuned the following parameters:

max depth This is the maximum depth that any DT can have. We test for $2, 4, 10$ or no restriction at all.

min samples leaf This is the minimum fraction of data points that must be allocated to any of the terminal leaves. We test for $2\%, 10\%$ or no restriction at all.

Another aspect that is often considered when working with individual decision trees is so-called post-pruning, whereby the trained decision tree is cut back to reduce prediction error on cross-validation data. In order to stay consistent and comparable with the other methods, here we do not apply such post-pruning. Instead, we use the cross-validation data to restrict the tree depth via the two hyperparameters above. This is in line with the approach of Esmeir and Markovitch [2], who argue that pruning the tree will make it difficult to compare search spaces between pruned and non-pruned trees. Further, pruning is unable to recover from wrong decisions, which makes it difficult to tackle the XOR problem that we focus on here.

XGB Hyperparameters

For XGBoost (XGB), the following parameters were tuned:

max depth This is the maximum depth that any DT can have. We test for $2, 4, 10$ or no restriction at all.

min samples leaf This is the minimum number of data points that must be allocated to any of the terminal leaves. We test for $2\%, 10\%$ or no restriction at all.

learning rate The boosting learning rate, which makes the model more robust by shrinking the weights on each step. We select this value as either $0.01, 0.05, 0.1$ or 0.2.

SI Section 3: Selection of Hyperparameters

In this section, we show the selection of the hyperparameters for all three experiments: synthetic data, commodity data and copper trading strategy. All parameters have been determined by means of a 5-fold cross-validation. The resulting selection is most easily visualized for the commodity data, because each asset has one set of hyperparameters. This is shown in SI-Table I.

SI-Table I: Selected hyperparameters across assets and tree algorithms.

asset	hyperparameter	LRF	GRF	GDT	XGB	asset	hyperparameter	LRF	GRF	GDT	XGB
coco	learning rate	x	x	x	0.2	natural gas	learning rate	x	x	x	0.01
	max depth	2	4	None	10		max depth	2	None	None	2
	max features	8	8	x	x		max features	8	8	x	x
	min samples leaf	0.1	None	None	0.1		min samples leaf	0.1	0.1	None	0.02
coffee	learning rate	x	x	x	0.2	oats	learning rate	x	x	x	0.05
	max depth	2	2	None	2		max depth	2	None	None	2
	max features	4	4	x	x		max features	4	4	x	x
	min samples leaf	0.1	None	0.1	0.1		min samples leaf	0.02	None	0.1	0.1
copper	learning rate	x	x	x	01	palladium	learning rate	x	x	x	0.05
	max depth	2	2	8	4		max depth	2	None	8	2
	max features	8	4	x	x		max features	4	8	x	x
	min samples leaf	None	None	None	None		min samples leaf	0.1	0.1	0.1	None
corn	learning rate	x	x	x	0.2	platinum	learning rate	x	x	x	0.01
	max depth	2	2	None	2		max depth	2	4	None	2
	max features	4	4	x	x		max features	8	8	x	x
	min samples leaf	None	None	None	None		min samples leaf	None	0.1	None	None
cotton	learning rate	x	x	x	0.1	rice	learning rate	x	x	x	0.1
	max depth	2	2	None	2		max depth	2	4	None	2
	max features	4	8	x	x		max features	4	4	x	x
	min samples leaf	None	0.02	None	None		min samples leaf	0.02	None	None	0.02
crude oil	learning rate	x	x	x	0.2	silver	learning rate	x	x	x	0.1
	max depth	2	2	4	4		max depth	2	2	8	2
	max features	4	4	x	x		max features	8	4	x	x
	min samples leaf	None	None	0.1	None		min samples leaf	0.02	0.1	None	0.1
gold	learning rate	x	x	x	0.2	soybean	learning rate	x	x	x	0.01
	max depth	2	4	None	2		max depth	2	2	10	2
	max features	4	8	x	x		max features	4	4	x	x
	min samples leaf	0.1	0.02	None	None		min samples leaf	None	None	0.02	0.1
heating oil	learning rate	x	x	x	01	sugar	learning rate	x	x	x	0.01
	max depth	2	2	None	2		max depth	2	2	None	2
	max features	4	4	x	x		max features	8	4	x	x
	min samples leaf	0.02	0.02	0.1	0.02		min samples leaf	None	0.02	0.1	None

For the synthetic data experiment, visualization of the selected hyperparameters is more tricky. For three different feature patterns (XOR and noise, XOR and linear, linear only), we have run $M = 20$ different tests for each of the 30 levels of ρ. To simplify the visualization, we show in Figures SI 3 - SI 5 the distribution of selected hyperparameters.

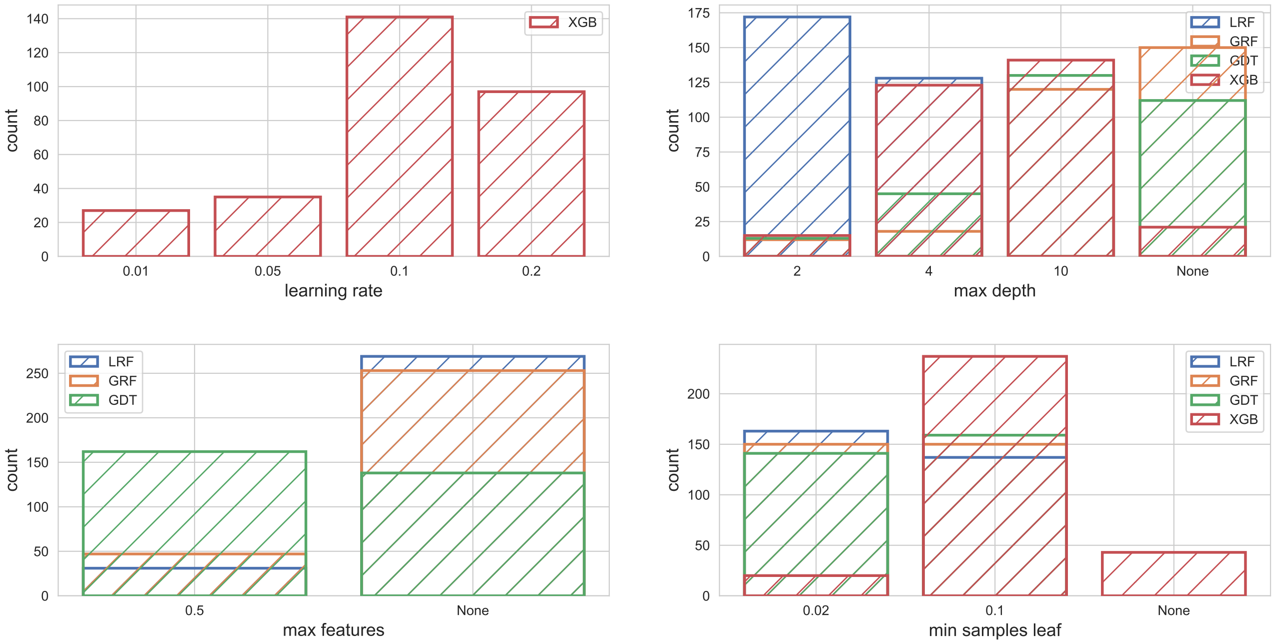

Figure SI 3: Each subplot shows the distribution of hyperparameters for the experiment with synthetic data for two XOR features and six random noise features. These are the hyperparameters that correspond to the experiment shown in Figure 2 of the main paper.

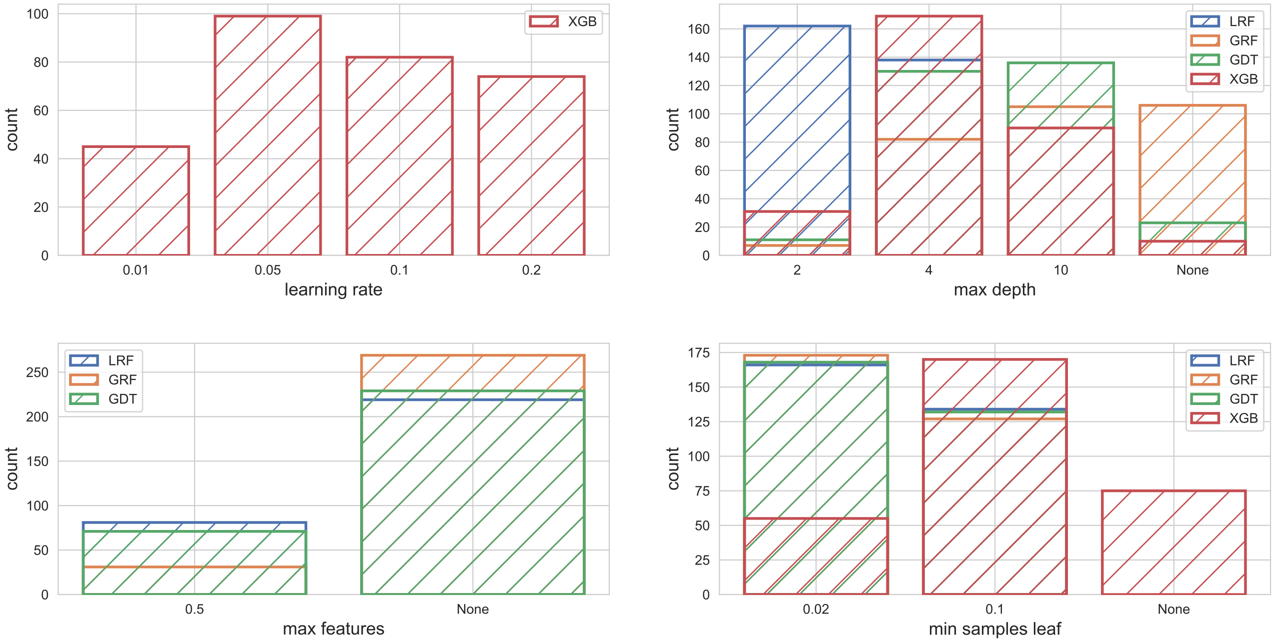

Figure SI 4: Each subplot shows the distribution of hyperparameters for the experiment with synthetic data for two XOR features and six linearly correlated features. These are the hyperparameters that correspond to the experiment shown in Figure SI 1.

For the trading strategy executed on copper futures, we have one additional hyperparameter to track: the signal execution threshold θ (cf. also below for details on this parameter). Since we retrain the model each 75 trading days from 2012 through 2020, we end up with roughly 90 different models. Rather than listing the individual hyperparameters in a table for each of the 90 time

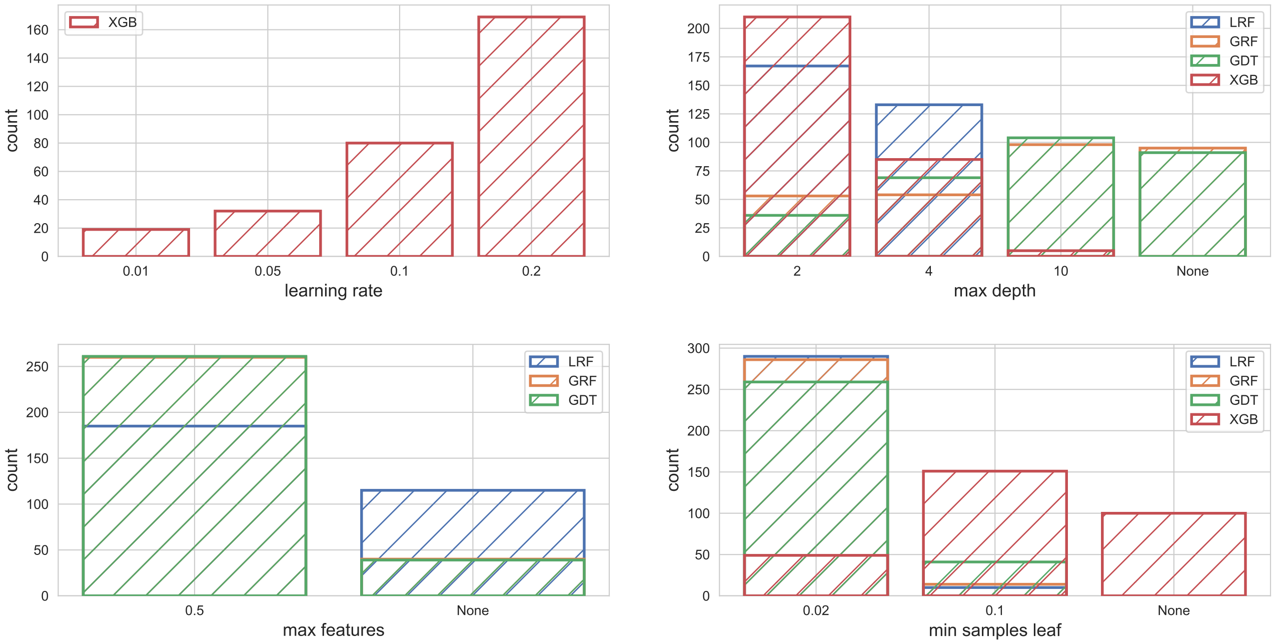

Figure SI 5: Each subplot shows the distribution of hyperparameters for the experiment with synthetic data for two XOR features and six linearly correlated features. These are the hyperparameters that correspond to the experiment shown in Figure SI 2.

periods, we instead show their distribution in Figure SI 6 and Figure SI 7. Figure SI 6 shows the distribution of hyperparameters across strategies with all 8 features. Figure SI 7 shows the distribution of hyperparameters across strategies with only 2 features.

SI Section 4: Asset Selection

When picking an asset to investigate the performance of the LRF relative to other classifiers, we must be careful to not try a myriad of assets at random until one (essentially out of luck) returns the desired results. With this in mind, we first narrow the eligible universe by eliminating

(A) all indices and instruments that do not include volume data,

(B) all assets and ETF's that split or pay dividends,

(C) all assets that seem too illiquid to trade without impact,

(D) all assets that are so liquid that return prediction based on simple technical indicators seems out of reach.

These criteria already exclude some of the most common equities, major indices and foreign exchange. We thus settle for front month commodity future contracts, since they are conveniently available from Yahoo Finance. Data ranges from 2012 through 2020 was used for all, with the last 20% set aside as the final out of sample test. The selected commodities are the ones presented in Table 1 of the main paper. We then select copper for further scrutiny because it is the one for which both LRF and GRF unambiguously outperform the benchmark long-only prediction (i.e. predicting the majority class). All the data used in this study has been downloaded from YahooFinance and is available for free. Moreover, YahooFinance is a source of many types of financial timeseries besides commodity futures. These include different equities, indices, and cryptocurrencies, amongst others. It is recommended as a good resource for data for the interested reader who wishes to conduct their own further research on such financial timeseries.

SI Section 5: Technical Indicators

We describe the technical indicators implemented in the Results section.

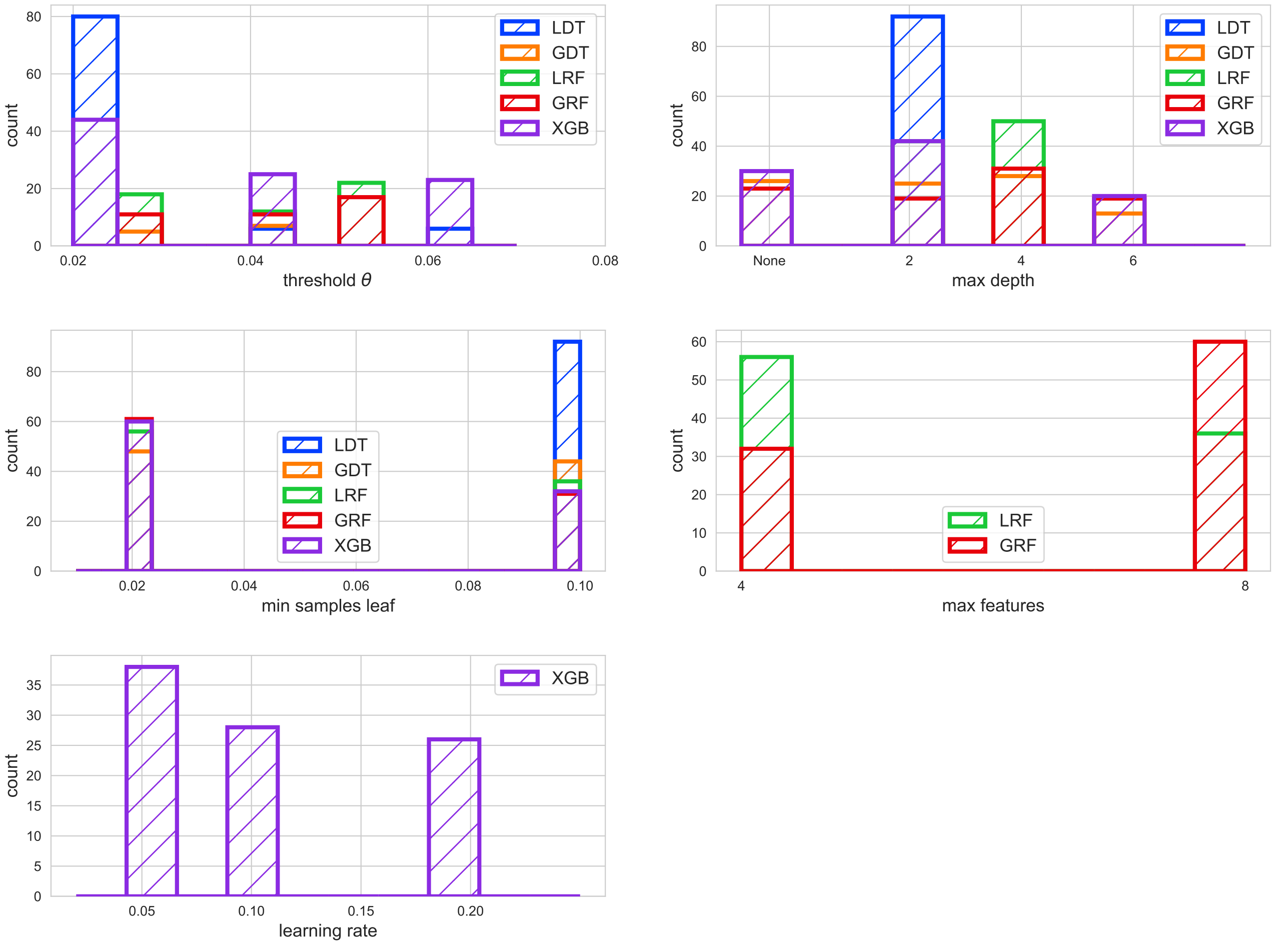

Figure SI 6: Each subplot shows the distribution of hyperparameters for the copper strategy. For each method, we have roughly 90 different trained instances, one for each rolling time window. Here, we show the results for strategies trained on all eight technical features. See also section 'More Copper Strategy Performances' below for details on the strategy performance.

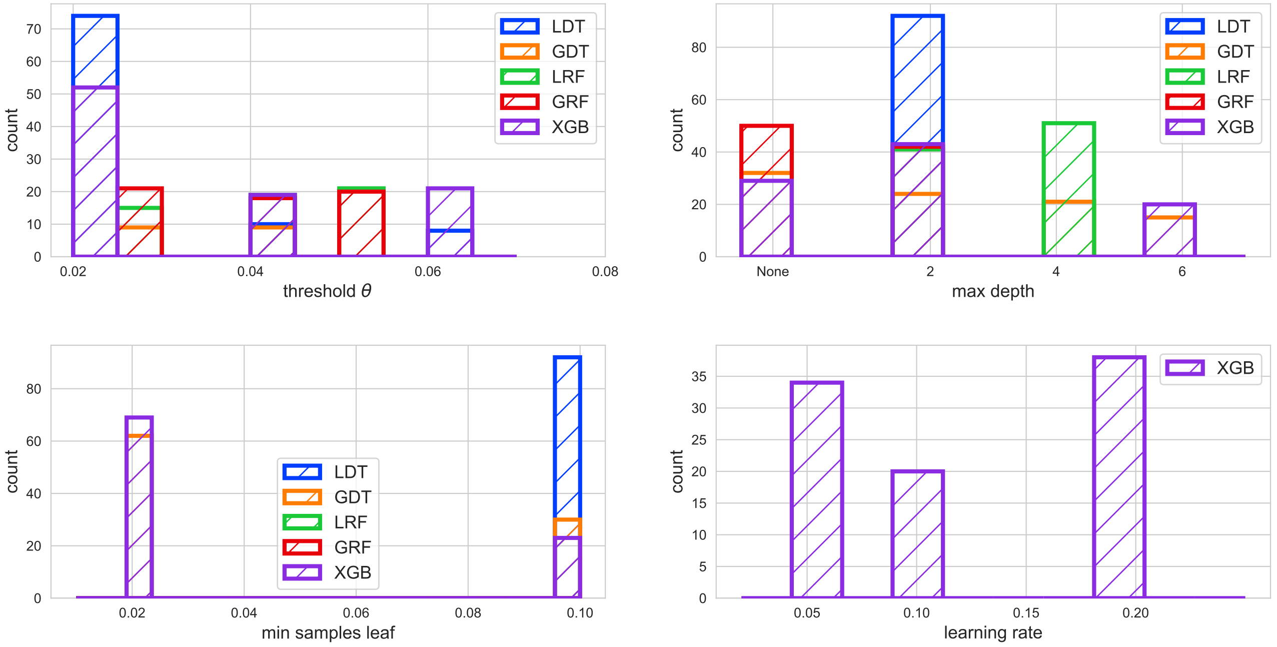

Figure SI 7: Same as Figure SI 6 but for the strategies trained with only two parameters.

- RSI (Relative Strength Index):
The RSI is a momentum indicator that tracks the recent price changes of an asset. It oscillates between 0 and 100. The 5-day and 20-day RSI's have been used in the Results section.

- Sign correlation:
This is the correlation coefficient between the past returns for n days. The correlation coefficient ranges from -1 to 1. The 5-day and 20-day Sign Correlations's have been used in the Results section.

- Overnight-gap:
This is the percentage difference between an asset's opening price and its previous day's closing price.

- Close location value:
This is used to measure the relative location of the closing price. It ranges in value from -1 to 1 and is calculated to measure the relative difference between an asset's closing price and its day's high and low prices.

- Volume-based Z-score:
This indicator is defined as the difference between an asset's volume and its trailing exponential moving average for the past n days divided by the standard deviation of the volume of the past n days. The 5-day and 20-day Volume-based Z-score's has been used in the Results section.

SI Section 6: Trading Strategy Extracted from the Return Sign Classifiers

In the results section, we extract a concrete trading strategy from the trained binary RF classifier that predicts the sign of daily copper returns. Prior to each (out of sample) trading day, the RF's $2,000$ trained DT's return a binary prediction - whether the following day's price return will be positive or negative. If, for instance, $1,400$ of these DTs predict that tomorrow's return will be positive, this translates to a signal strength of $1,400/2,000 = 70\%$, and so forth. For the following trading day, we are long/short the asset if the signal is $\theta\%$ above/below the neutral 50%. If it is not, we take no position for that trading day. The threshold θ is a hyperparameter to be selected along with the classifier hyperparameters. Its values are $1\%, 2.5\%, 4\%$ and 5%. Strategies must trade at least 20% of all eligible trading days to be considered. This may effectively remove some of the parameter combinations ex-ante. Performance is evaluate by means of the Sharpe ratio [3]. To extend a trading strategy over longer time periods, we repeat the above process in rolling windows. Here, we have considered rolling windows that consist of 500 trading days and are rolled in steps of 75 trading days. For a given window, we denote t_0 as the first trading day of the corresponding out-of-sample prediction dataset. Binary RF classifiers are trained on the previous 500 days, t_{-500} through t_{-1}. For cross-validation, these 500 data-points are split into folders of 500 datapoints each. To avoid leakage between folds due to serial correlation, we add embargo zones of 20 days, with 10 days on either side of the fold, effectively reducing the number of training data to 400 days. The eight basic technical indicators are described above. We further assume instantaneous trade execution and do not account for slippage or trading costs. These simplifications do not affect the analysis presented, since our objective is to evaluate the relative effectiveness of different classifiers, not to build out viable trading strategies.

The above selection of hyperparameters is obtained by means of 5-fold cross-validation. There is an important distinction between the synthetic data and the financial data. For the synthetic data, the datapoints have no temporal ordering and hence the folds may be selected by randomly selecting data. For the financial data, the features have temporal memory and hence subsequent datapoints (i.e. subsequent days) are partially correlated. Therefore, it is important to retain temporal order when selecting the folds. Our longest lookback features are built with 20 training days. Therefore, we also remove the first and last 10 days from each fold, to avoid any cross-correlation between the samples of different folds (so-called embargo zones [1]).

SI Section 7: More Copper Strategy Performances

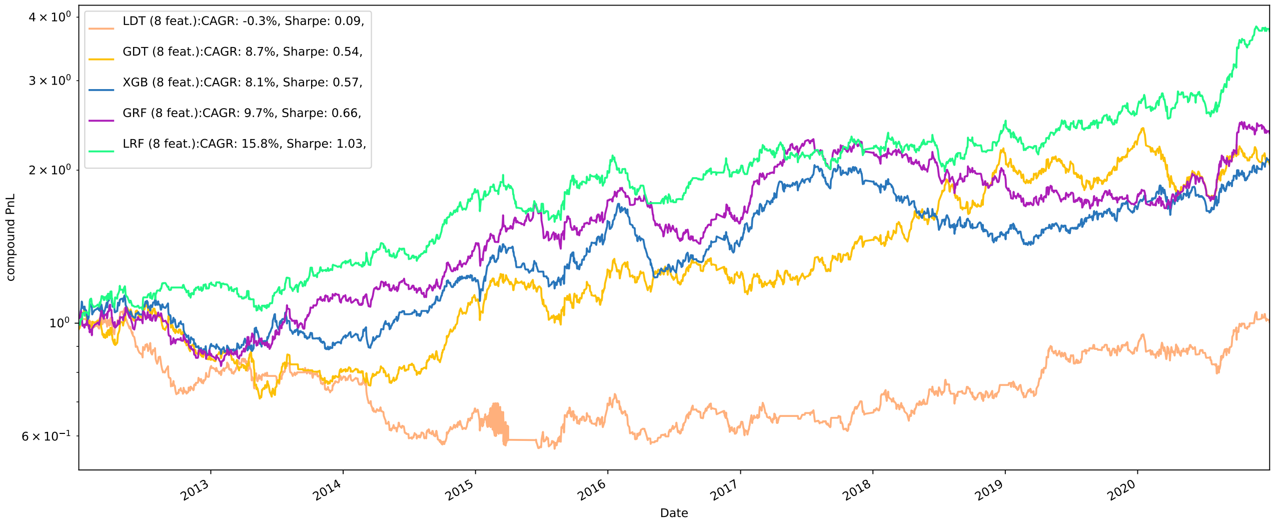

Figure SI 8: Copper strategy performance for different machine learning methods. All methods here are trained on the same eight technical features. The LRF and GRF strategies are equivalent to those represented by the continuous lines of Figure 3 in the main paper.

In Figure 3 of the main paper, we show the performance of the copper trading strategy described in the above section. We first train an LRF and a GRF on eight technical features. Subsequently, we isolate two features that exhibit an XOR-pattern, and train and trade purely on those. In the main paper, we have restricted our attention to the LRF and the GRF for simplicity. Here, we additionally show the performance of XGB, as well as LDT and GDT. Figure SI 8 visualizes the performance of all five methods when trained across all eight features. The LRF clearly outperforms the other methods. As the general degradation from eight features to two features in Figure SI 9 shows, there is value in more than just the two XOR-type features. The LDT with limited depth fails to take all these features into consideration, and hence its relative underperformance is no surprise. However, when only the two XOR-type features are used, the LDT outperforms all other methods, except for the LRF. We explain the underperformance of the LDT relative to the LRF when only two features are present as a result of bootstrapping. The resampling of the data and averaging across different forests yields a smoother trading signal. More generally, the low signal-to-noise ratio of the problem at hand calls for the application of decision forests, to average out the high variance.

References

[1] M. L. De Prado. *Advances in financial machine learning.* John Wiley & Sons, 2018.

[2] S. Esmeir and S. Markovitch. Lookahead-based algorithms for anytime induction of decision trees. In *Proceedings of the twenty-first international conference on Machine learning*, page 33, 2004.

[3] A. W. Lo. The statistics of Sharpe ratios. *Financial analysts journal*, 58(4):36–52, 2002.

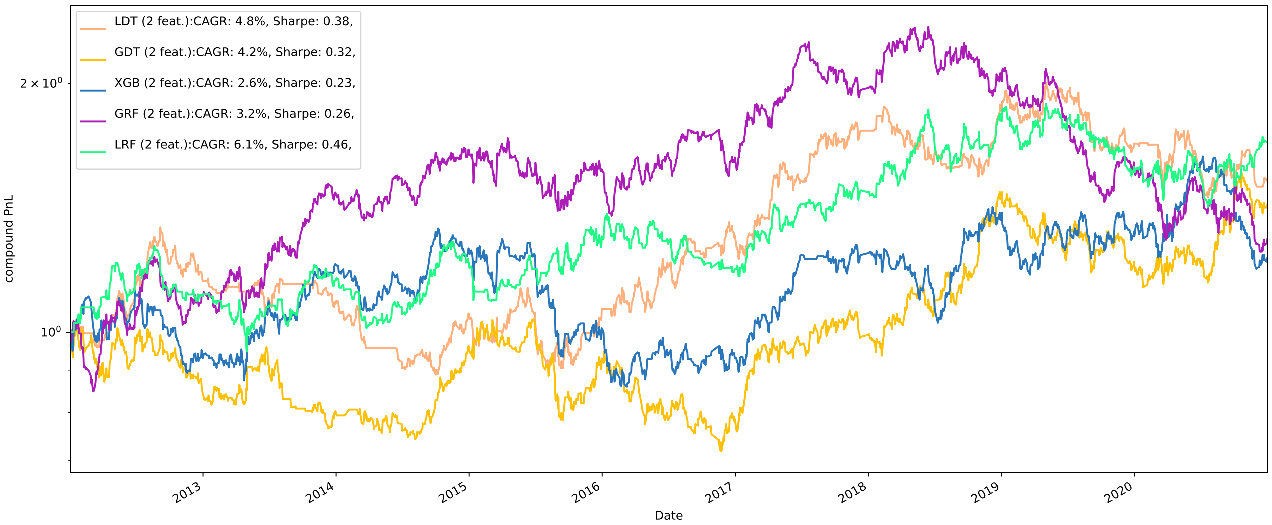

Figure SI 9: Copper strategy performance for different machine learning methods. All methods here are trained on only two technical features ('vol Z score 5' and 'RSI 20'). The LRF and GRF strategies are equivalent to those represented by the dashed lines of Figure 3 in the main paper.
